# Towards a Framework for Observational Causality from Time Series: When Shannon Meets Turing

**DOI:** 10.3390/e22040426

**Published:** 2020-04-09

**Authors:** David Sigtermans

**Affiliations:** ASML, De Run 6501, 5504 DR Veldhoven, The Netherlands; david.sigtermans@asml.com

**Keywords:** information theory, transfer entropy, time-delayed mutual information, data processing inequality, time series, causal tensor

## Abstract

We propose a tensor based approach to infer causal structures from time series. An information theoretical analysis of *transfer entropy* (*TE*) shows that *TE* results from transmission of information over a set of communication channels. Tensors are the mathematical equivalents of these multichannel causal channels. The total effect of subsequent transmissions, i.e., the total effect of a cascade, can now be expressed in terms of the tensors of these subsequent transmissions using tensor multiplication. With this formalism, differences in the underlying structures can be detected that are otherwise undetectable using TE or mutual information. Additionally, using a system comprising three variables, we prove that bivariate analysis suffices to infer the structure, that is, bivariate analysis suffices to differentiate between direct and indirect associations. Some results translate to TE. For example, a *Data Processing Inequality* (*DPI*) is proven to exist for transfer entropy.

## 1. Introduction

Many real-world phenomena are nonlinear and stochastic, and therefore difficult to model. Several methods have been developed over time to recover information from time-series characterizing these phenomena, many of which are parametric in nature. These parametric models assume certain relationships, e.g., linear relationships, between the random variables to reconstruct the (statistical) characteristics of the parameters of interest. The difficulty with this approach is that oftentimes the nature of their interaction is unknown, and that “*the whole is greater than the sum of its parts*”, i.e., the processes constituting such a system can behave synergistically.

**Figure 1:**
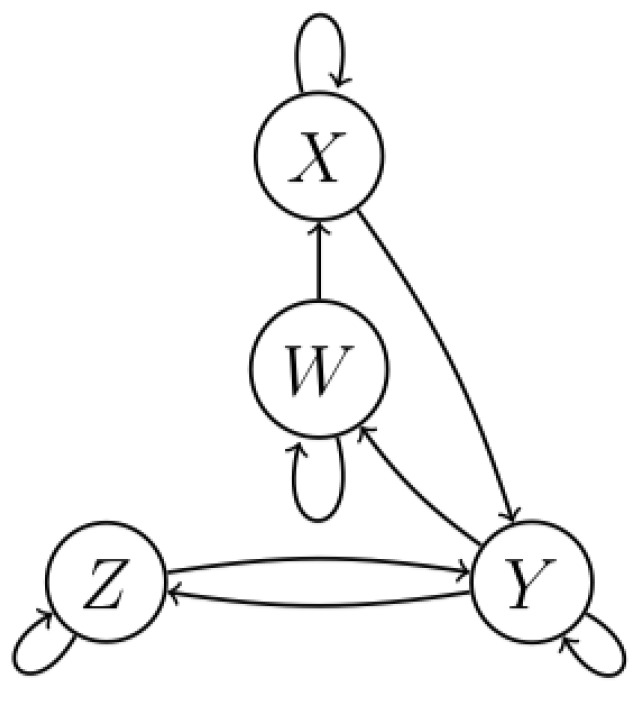
This graph represents the underlying (temporal) structure for a system of four linearly and non-linearly coupled Ornstein–Uhlenbeck processes [3]. The vertices represent random variables. The directed edges indicate the directionality of the association.

The underlying structure of complex systems can be represented by graphs [1] (see, for example, Figure 1). The variables representing underlying processes are the nodes; the directed edges indicate how the processes influence each other. Knowledge of this structure allows us to understand and predict the behavior of the complex system [2].

The existence, directionality, and strength of influences is the subject of causal inference [4]. In a causal relation, the cause precedes the effect, and the cause physically influences the effect [5]. A causal description is essentially different from a description via statistical associations as illustrated by the adage “*correlation does not imply causation*”. Apart from directionality of associations, a causal description can differentiate between direct associations and indirect associations (an indirect association is an association via one or more mediators). To differentiate unequivocally between direct and indirect associations, intervention is required [4]. Since interventions, that is, experiments, are not always possible, we have to make do with observational data. A plethora of nonparametric methods to infer causal structures from observational data have been developed (see, for example, [3,6,7,8,9,10,11,12,13,14]). What most of these methods have in common is that they describe associations via pointwise estimators representing the “strength” of the association. To differentiate direct from indirect associations, these methods all use multivariate analysis, leading them to suffer from the “curse of dimensionality” [3].

In this article, we propose a formalism that is inspired by Turing machines [15] and the success of one of the nonparametric measures, transfer entropy [10,16,17]. If a human “computer” can decide, given the data, that a relation is causal, a Turing machine exists that reaches this decision in a mechanical way [18]. This is not a tautology, a Turing machine encodes the underlying principles leading to the decision that a relation is causal in the “transition function”. To determine this transition function for transfer entropy, information theory [19] is used. This theory reflects the idea that meaningful communication can only exist if there is an association between the data sent, the source data, and the data received, the destination data. The amount of association between the data is determined by characteristics of the *communication channel* over which data are transmitted. These characteristics, the transition probabilities, can be expressed in stochastic matrices [20], or, more generally, in stochastic tensors [21], i.e., multilinear maps.

For observational data in general and time series specifically, these tensors can be determined for all source/destination pairs. The resulting tensor of a sequence of consecutive *direct paths*, for example, the cascade X→Y→Z in Figure 1, can be expressed in terms of the tensors of the constituting direct paths X→Y and Y→Z, respectively. Apart from using this to derive a data processing inequality [20] for transfer entropy, it enables differentiating between direct and indirect associations using bivariate analysis. The proposed formalism allows us to decide on the existence and the directionality of associations using observational data, via bivariate analysis.

The rest of this article is organized as follows. The proposed formalism is based on probability theory [22] and causal inference [4]. In Section 1.1, the most relevant aspects are summarized. The applicable aspects of information theory are presented in Section 2.1. In Section 2.2, transfer entropy (TE) is introduced, and it is shown that it is the average mutual information resulting from transmission of data via a network of communication channels with a very specific topology: the *multichannel causal channel*. Using these multichannel causal channels instead of a scalar representing the “strength” of an edge, the resulting tensor of a path comprising two or more edges can now be expressed in terms of the tensors of the constituting edges. In Section 3, we show that this more specific description of edges allow us to differentiate between direct and indirect associations, and that bivariate analysis suffices.

For readability, we move the proposed definitions, theorems, and the proofs to the Appendix A.

### 1.1. Preliminaries

**Figure 2:**
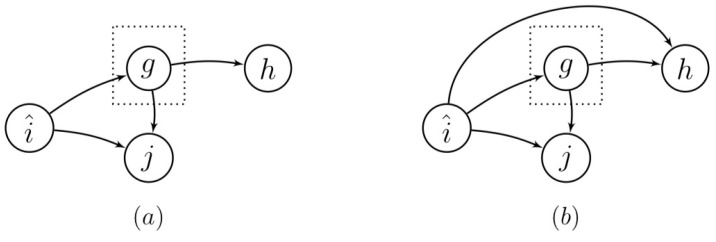
Illustration of the Causal Markov Condition. (**a**) The vertex in the dotted box blocks all paths between $\hat{i}$ and *h*, and between *j* and *h*. According to the Causal Markov Condition, $\left\{\hat{i},j\right\}$ and *h* are independent given *g*: $p\left(\hat{i},j,h|g\right)\;=\;p\left(\hat{i},j|g\right)p\left(h|g\right)$. (**b**) The vertex in the dotted box does not block all path between $\hat{i}$ and *h*, and between *j* and *h*. According to the Causal Markov Condition, $\left\{\hat{i},j\right\}$ and *h* are *not* independent given *g*, i.e., $p\left(\hat{i},j,h|g\right)\;\neq \;p\left(\hat{i},j|g\right)p\left(h|g\right)$.

Statistical independence is foundational to causal inference [9]. The two most relevant assumptions are: (1) the Causal Markov Condition; and (2) the faithfulness assumption. The Causal Markov Condition states that a process is independent of its non-effects given its direct causes or its parents. This is relevant in the context of time series. A straightforward interpretation of this condition is that, if a set of variables blocks all (undirected) paths between two variables, these two variables are independent given the set of variables blocking all paths [4] (see Figure 2).

A directed graph is said to be faithful to the underlying probability distributions if the independence relations that follow from the graph are the same independence relations that follow from the underlying probability distributions. For the chain $\hat{i}\to g\to h$ in the graph of Figure 2a, the faithfulness assumption implies that $\hat{i}$ and *h* are independent given *g*. This is denoted as $\hat{i}⊥\;⊥h|g$.

In the example above, we use a simplified notation for the probabilities p(y):=p(Y=y). Furthermore, basic aspects of probability theory such as the Law of Total Probability [22] are used. This law links a marginal probability to a joint probability, e.g., $\sum_{g}p\left(j,g|i,h\right)\;=\;p\left(j|i,h\right)$. Unless stated otherwise, the Einstein summation convention is used. This convention simplifies equations by implying summation over indices that appear both as upper indices and as lower indices, e.g, $p\left(j,g|i,h\right))\;:=\;\sum_{g}p\left(j,g|i,h\right))$. The summation is taken over the upper indices and the subsequent identical lower indices. This implies that $B_{j}^{i}A_{i}^{j}\neq A_{i}^{j}B_{j}^{i}$, the order matters.

In this article, a chain, cascade, or “path” is considered equivalent and used interchangeably. For example, the chain, or cascade X→Y→Z, represents a “transmission path”, that is, the sequence in which vertices are used to transmit the data. A path is represented as path{source, $mediator_{1},\cdots ,mediatior_{n},destination\}$. The chain X→Y→Z represents the path{X,Y,Z}.

## 2. Materials and Methods

### 2.1. Information Theory

Information theory was introduced in 1948 by C. Shannon [19]. It is based on the idea that meaningful communication can only exist if there is an association between the message sent, and the message received. The amount of association is determined by the characteristics of the medium used to transmit the message, i.e., the communication channel. Information theory is a mathematical description of this communication process. A message comprises random variables representing stationary ergodic processes. An input message is first encoded: we describe the message using a finite alphabet. Each random variable has its own finite alphabet. The random variable *X* selects symbols from the alphabet X, the random variable *Y* selects symbols from Y, and the random variable *Z* is selects symbols from Z. Here, $X\;=\;\{\chi_{1},\chi_{2},\cdots ,\chi_{\left|X\right|}\}$, $Y\;=\;\{\psi_{1},\psi_{2},\cdots$, $\psi_{\left|Y\right|}\}$, and $Z\;=\;\{\zeta_{1},\zeta_{2},\cdots ,\zeta_{\left|Z\right|}\}$. The number of elements in the alphabet, the cardinality, is denoted as |X|, |Y|, and |Z|, respectively. Once encoded, the message is transmitted symbol by symbol via a so-called communication channel. The input symbol is transformed into an output symbol due to specific characteristics of the communication channel. The transmitted message is decoded and made available to the receiver. In this article, we assume that no decoding takes place. Due to this transmission, there is a certain amount of association between the two messages: information is said to be shared between them. The amount of information shared,

(1) $$I\left(X;Y\right)=\sum_{x\in X,y\in Y}p\left(x,y\right)\log_{2}\left[\frac{p(y|x)}{p(y)}\right],$$

is nonnegative and symmetric in *X* and *Y* [20]. This so-called mutual information (MI) represents the reduction in uncertainty about the random variable *Y* given that we have knowledge about the random variable *X* (and vice versa). Mutual information is nonparametric and capable of capturing nonlinearity [23]. It is intuitively clear that, given the information content of the source, data can never increase due to subsequent transmissions. This is formalized in the data processing inequality (DPI), which states that processing of data can never increase the amount of information [20]. For the cascade X→Y→Z, the DPI implies that, in terms of MI,

I(X;Z)≤min[I(X;Y),I(Y;Z)].

The maximum rate with which information can be transmitted between the sender and receiver is the channel capacity $C_{XY}\;=\;\max_{p(x)}\left[I(X;Y)\right]$ [20]. This is achieved for a so-called channel achieving input distribution.

#### 2.1.1. The Communication Channel

A communication channel is modeled as a Markov chain. The channel has an input side—the left-hand side—and an output side—the right-hand side. On the left-hand side, all the vertices of the Markov chain with outgoing edges are drawn, and, on the right-hand side, all the vertices of the Markov chain with incoming edges are drawn. The input vertices are connected to the output vertices via undirected edges. In a channel, every input symbol has its own input vertex. Likewise, every output symbol has its own output vertex. The probability that a specific output symbol is received only depends on the alphabet symbol that was sent. The communication process transforms the input probability mass function (pmf) into the output pmf. This transformation is specific to the communication channel.

The simplest type of channel is the noisy discrete memoryless communication channel (DMC). In a memoryless channel, the output, $y_{t}$, only depends on the input, $x_{t}$, and not on the past inputs or outputs: $p\left(y_{t}|x_{t},x_{t-1},y_{t-1}\right)\;=\;p\left(y_{t}|x_{t}\right)$. A memoryless channel embodies the Markov property. In a noisy channel, the output depends on the input and another random variable representing noise. The more noise there is, the less association there is between the source and destination message. The effect of transmitting data using a DMC is described via the Law of Total Probability because

(2) $$p\left(Y=\psi_{j}\right)=\sum_{i}p\left(X=\chi_{i}\right)p\left(Y=\psi_{j}|X=\chi_{i}\right),$$

with $p(Y\;=\;\psi_{j})$ the *j*th element of the probability mass function p(y), and $p(X\;=\;\chi_{i})$ the *i*th element of the pmf p(x). The transmission of data over a DMC transforms the pmf of the input into the pmf of the output via a linear transformation. The probability transition matrix $p(Y=\psi_{j}|X=\chi_{i})$ fully characterizes the DMC [20]. Assuming a fixed (e.g., lexicographic) order of the alphabet elements, we can introduce an index notation for the pmfs, e.g., $p^{j}\;:=\;p\left(Y\;=\;\psi_{j}\right)$ and $p^{i}\;:=\;p\left(X\;=\;\chi_{i}\right)$. In this article, each random variable has its own, fixed set of associated indices. In Table 1, an overview is given.

#### 2.1.2. Tensor Representation of the Communication Channel

The channel transforms a source symbol via a linear mapping into a destination symbol, i.e., the source probability density function is mapped onto the destination pmf via a probability transition matrix [20]. With $p^{j}$ representing the *j*th element of the destination pmf, $p^{i}$ representing the *i*th element of the source pmf, and $p_{i}^{j}\;=\;p\left(Y=\psi_{j}|X=\chi_{i}\right)$, the relation between source and destination is given by the matrix multiplication $p^{j}\;=\;\sum_{i}p^{i}p_{i}^{j}$ [24]. Because a matrix is specific to a communication channel, it makes sense to identify each channel and its probability transition matrix by one and the same “name”, for example, $p_{i}^{j}:=A$. Using the earlier mentioned Einstein summation convention, the linear transformation of the source pmf into the destination pmf can be written as

(3) $$p^{j}=p^{i}A_{i}^{j}.$$

The covariant or lower indices indicate the variables we condition on. The row stochastic probability transition matrix elements represent the elements of the probability transition tensor A [21]. Because the summation in Equation (1) is performed over the alphabet elements for *x* and *y*, MI can also be written in terms of these indices. With $p\left(x,y\right)\;=\;p^{ij}$ and using the standard notation instead of the Einstein summation convention, Equation (1) therefore equals

(4) $$I\left(X,Y\right)=\sum_{i,j}p^{ij}\log_{2}\left[\frac{A_{i}^{j}}{p^{j}}\right].$$

Assuming that the structure is independent of the input, MI is not an optimal measure to infer the underlying structure because it depends on both the tensor, and on the input pmf. Even if the channel is noiseless, the MI could be negligible because of the input pmf. This is illustrated in the following example.

**Example** **1.** *Let us assume that the probability transition tensor equals the Kronecker delta [24]*

$$\delta_{i}^{j}=\left\{\begin{array}{cc} 1, & \mathrm{if}\;i=j,\\ 0, & \mathrm{if}\;i\neq j. \end{array}\right.$$

*If $A_{i}^{j}\;=\;\delta_{i}^{j}$, the symbol received is identical to the symbol sent; the channel transmits data perfectly. In this case, MI reduces to $I\left(X;Y\right)=\sum_{i}p^{i}\log_{2}\left[1/p^{i}\right]$. Now, set the probability of one of the alphabet elements to 1−ε. This implies that all other symbol probabilities are equal to or smaller than ε. Taking the limit ε→0 results in a mutual information →0. Although there might be a noiseless channel representing the association between the random variables X and Y, MI could be arbitrarily small.*

This example illustrates that the absence of association does not negate the existence of an underlying pathway, nor does it negate data transmission: data are transmitted. By simply tweaking the input pmf, we can make the association arbitrarily small while still transmitting data. Instead of inferring the *structure* using MI or MI related measures, the probability transition tensors should be used. Because the earlier mentioned channel capacity only depends on the elements of the tensors [25], the maximal potential association can always be determined.

In this short and incomplete introduction to information theory, no assumptions, other than stationarity, ergodicity, and Markov property, were made about the underlying mechanisms leading to the association between random variables. We can therefore apply information theory to all cases where observational data satisfy these assumptions. However, due to the symmetry in *X* and *Y*, mutual information cannot differentiate a source from a destination. To introduce an asymmetry, time-delayed mutual information has been proposed (see, for example, [7]). It uses the asymmetric temporal relation between a cause and it effect. The time delay that optimizes the MI is the “interaction delay”. Unfortunately, time delayed mutual information does not satisfy “Wieners principle of causality” [26]: a cause combined with the past of the effect predicts the effect better than that the effect predicts itself. This principle is foundational to the well known and much used Granger Causality [27].

### 2.2. Transfer Entropy

In 2000, transfer entropy (TE) was introduced [10], an information theoretical implementation of “Wieners principle of causality”. Similar to mutual information, it is non-parametric, but, unlike MI, it is an essentially asymmetric measure and it enables the differentiation between a source and a destination. It was proven that, with a slight modification of the original proposed TE, transfer entropy fully complies with Wieners principle of causality [28]. Because TE is based on information theory, it is nonparametric and capable of detecting nonlinear relationships (see, for example, [29]). This modified TE can recover interaction delays: it is maximal for the real interaction delay τ. With $x^{-}$ representing the cause, $y^{-}$ the past of the effect, and *y* as the effect,

(5) $$TE_{X\to Y}=\sum_{\begin{array}{c} x^{-}\in X^{m},y\in Y\\ y^{-}\in Y^{\ell } \end{array}}p\left(x^{-},y,y^{-}\right)\log_{2}\left[\frac{p(y|x^{-},y^{-})}{p(y|y^{-})}\right].$$

It is assumed that *Y* is a Markov process of order ℓ≥1, i.e., the effect *y* also depends on its own past $y^{-}\;=\;\left(y_{t-1},\cdots ,y_{t-\ell }\right)$. If Y represents the alphabet of *Y*, then the alphabet for the past of *Y* is given by $Y^{\ell }$. It is furthermore assumed that the destination depends on a vector of source symbols, $x^{-}\;=\;\left(x_{t-\tau },\cdots ,x_{t-\tau -m}\right)$, with m≥0. If X represents the alphabet of *X*, then the alphabet for the input is given by $X^{m}$.

Transfer entropy is asymmetric in *X* and *Y*, and therefore capable of distinguishing a source from a destination. To differentiate a source from a destination, we have to assess two hypotheses: (1) *X* is the source and *Y* is the destination; and (2) *Y* is the source and *X* is the destination. Per hypothesis, the interaction delay that maximizes the respective TE is determined. There are three possibilities. The first possibility is that neither of the two associations differ significantly from zero: there is no association. In case both associations differ significantly from zero, a cycle is assumed to exist. The third possibility is that only one association differs significantly from zero.

When using TE to infer the underlying structure in a complex system, as depicted in Figure 1, three fundamental issues will be encountered. The first issue arises when data are transmitted along a *path* comprising over two nodes. For example, in the case of Figure 1, the transmission from the source *x* to the destination *w* via the mediator *y*, i.e., transmission along the path{x,y,w}. In this case, the resulting TE cannot be expressed in terms of the transfer entropies of the constituting transmissions.

The second issue is related to indirect associations. In the original, bivariate definition of TE, it is not possible to distinguish between direct and indirect associations using TE. The transfer entropy between *z* and *x* in Figure 1 would be larger than zero, i.e., without further information we would draw an edge to reflect the existence of the path{x,z}. However, if the association between two vertices is indirect, there does not exist a direct path between these nodes as per the faithfulness assumption. To resolve this issue, multivariate approaches have been developed. These methods are computationally expensive and suffer from the above-mentioned curse of dimensionality.

The third issue is that TE, similar to MI, can be made arbitrarily small due to a specific choice of the input data. It is therefore not an optimal measure to infer structures. All three issues can be resolved when reverting to the generating process resulting in TE as a measure of association: the communication process.

#### 2.2.1. The Causal Channel

Transfer entropy is a conditional mutual information [10]. Therefore, it can be associated with communication channels. Conditioning the mutual information of Equation (1) on $y^{-}\;=\;\psi_{g}^{-}$ results in

(6) $$I\left(X;Y|y^{-}\;=\;\psi_{g}^{-}\right)=\sum_{\begin{array}{c} x^{-}\in X^{m}\\ y\in Y \end{array}}p\left(x^{-},y|y^{-}\;=\;\psi_{g}^{-}\right)\log_{2}\left[\frac{p(y|x^{-},y^{-}\;=\;\psi_{g}^{-}))}{p(y|y^{-}\;=\;\psi_{g}^{-})}\right].$$

The reader can verify that transfer entropy, Equation (5), can now be written as

(7) $$TE_{X\to Y}=\sum_{\psi_{g}^{-}\in Y^{\ell }}p\left(\psi_{g}^{-}\right)I\left(X;Y|y^{-}\;=\;\psi_{g}^{-}\right).$$

**Figure 3:**
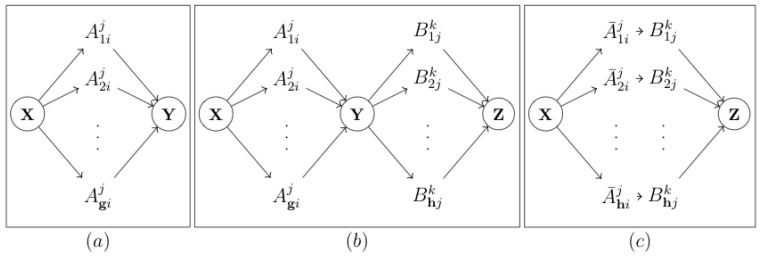
(**a**) The inverse multiplexer representing the communication network between *X* and *Y*. Source data are partitioned on the past of the effect and transmitted via the related communication channel. (**b**) The inverse multiplexers representing the transmission path{x,y,z}. (**c**) An equivalent representation network communication channels representing the transmission path{x,y,z}.

Because $x^{-}$ and $y^{-}$ in Equation (6) are the only parents of the output *y*, it follows from the Causal Markov Condition that the associated channel is memoryless. Equation (6) therefore quantifies the amount of information that is transmitted over the DMC of the *g*th sub-channel. Transfer entropy results from data transmission via a network of communication channels with the topology of an inverse multiplexer [30]. An inverse multiplexer comprises a demultiplexer and a multiplexer in series (see Figure 3a). The demultiplexer selects the sub-channel over which the data are send based on the past of the output data. Each sub-channel comprises a DMC. The input symbol is fed to a specific input vertex of the chosen discrete memoryless channel. The DMC transforms the input in a probabilistic fashion into an output symbol. The multiplexer combines the outputted symbols into the output message. We call this channel a *causal channel*.

**Definition** **1** (Causal Channel)**.** *A causal channel is an inverse multiplexer in which the demultiplexer selects the sub-channel over which the data are sent based on the past of the output data. Each sub-channel comprises a DMC. The input symbol is fed to a specific input vertex of the chosen discrete memoryless channel. The DMC transforms the input in a probabilistic fashion into an output symbol. The multiplexer combines the outputted symbols into the output message.*

This leads to the central theorem of our formalism.

**Theorem** **1.** *Transfer entropy is the average conditional mutual information of transmission over a causal channel.*

**Proof.**  The relative frequency with which the *g*th sub-channel is chosen equals $p(\psi_{g}^{-})$. Each sub-channel is a DMC, so the mutual information of the *g*th sub-channel equals $I(X;Y|\psi_{g}^{-})$. The weighted average of the mutual information over all the sub-channels is equal to $\sum_{\psi_{g}^{-}\in Y^{\ell }}p\left(\psi_{g}^{-}\right)I\left(X;Y|\psi_{g}^{-}\right)$, which is the definition of TE in Equation (7). □

A DMC is a causal channel with only one sub-channel, we therefore call a DMC a *mono-channel causal channel*. A causal channel with multiple sub-channels is called a multichannel causal channel. Every sub-channel can be represented by a probability transition matrix. Therefore, a multichannel causal channel can be represented by a probability transition tensor, the *causal tensor*. This stochastic tensor transforms the (conditional) input pmf into the output pmf. Using Equation (3) and conditioning all probabilities on *g*, the transformation for the *g*th sub-channel for the relation X→Y is given by

(8) $$p_{g}^{j}=p_{g}^{\hat{i}}A_{g\hat{i}}^{j},$$

with $A_{g\hat{i}}^{j}\;=\;p\left(\psi_{j}|\chi_{\hat{i}}^{-},\psi_{g}^{-}\right)$ the elements of the tensor A. Because the input could be a vector, $\hat{i}$ is used instead of *i* (see Table 1). Analogous to mutual information (see Equation (4)), TE can be rewritten as

(9) $$TE_{X\to Y}=\sum_{g,\hat{i},j}p^{\hat{i}jg}\log_{2}\left[\frac{A_{g\hat{i}}^{j}}{p_{g}^{j}}\right].$$

**Figure 4:**
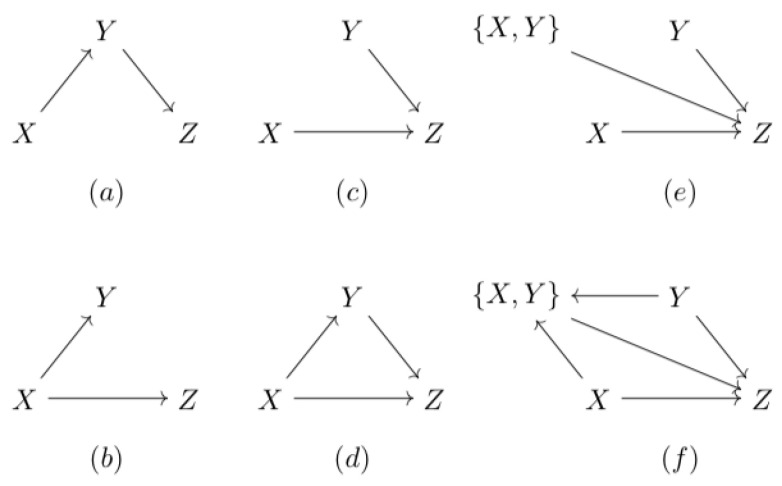
The basic structures directed graph structures: (**a**) the chain; (**b**) the fork; (**c**) the v-structure; and (**d**) the directed triangle. (**e**,**f**) The graphs reflect the calculation rules for the causal tensors for the v-structure and directed triangle, respectively.

Instead of describing an edge with its strength, we propose to use the tensors themselves. Using the proposed tensor formalism, we are now able to derive specific expressions for the resulting tensors of the three basic structures depicted in Figure 4a–c, i.e., the chain, the fork, and the v-structure.

#### 2.2.2. The Chain

In the case of a bivariate analysis of the chain X→Y→Z, one would expect to measure three associations and their related tensors: A:X→Y, B:Y→Z and C:X→Z. For two DMCs, i.e., two mono-channel causal channels in a cascade, the resulting transition probability matrix results from the matrix multiplication of the two constituting matrices (see, for example, [31]). The cascade of the related inverse multiplexers is depicted in Figure 3b. In Appendix A.2, a formal proof is given, but, using this figure, one can intuit that the tensor elements of the indirect association C are given by

(10) $$C_{h\hat{i}}^{k}=p_{h\hat{i}}^{g}A_{g\hat{i}}^{\hat{j}}B_{h\hat{j}}^{k}.$$

Let us for example backtrack the input for, say, the sub-channel with tensor elements $B_{1j}^{k}$. *Given* that this sub-channel is chosen for the *second* transmission, let us consider the contribution of the *first* transmission via, say, the sub-channel $A_{2\hat{i}}^{j}$. The contribution of the first transmission via this sub-channel *given* that $B_{1j}^{k}$ is used for the *second* transmission, equals the total contribution of sub-channel $A_{2\hat{i}}^{j}$, multiplied by the probability that this sub-channel is chosen, $p_{1\hat{i}}^{2}$. Because $p_{h\hat{i}}^{g}A_{g\hat{i}}^{\hat{j}}$ is a stochastic tensor (see Appendix A.2), Equation (10) can be rewritten as

(11) $$C_{h\hat{i}}^{k}={\bar{A}}_{h\hat{i}}^{\hat{j}}B_{h\hat{j}}^{k},$$

with

(12) $${\bar{A}}_{h\hat{i}}^{\hat{j}}\;=\;p_{h\hat{i}}^{g}A_{g\hat{i}}^{\hat{j}}.$$

Using this tensor product, an exact expression for the transfer entropy resulting from transmission over the path{X,Y,Z} can now be given:

(13) $$TE_{X\to Z}=\sum_{\hat{i},h,k}p^{\hat{i}hk}\log_{2}\left[\frac{\sum_{\hat{j}}{\bar{A}}_{h\hat{i}}^{\hat{j}}B_{h\hat{j}}^{k}}{p_{h}^{k}}\right].$$

Please note that no exact expression exists for $TE_{X\;\to \;Z}$ in terms of $TE_{X\;\to \;Y}$ and $TE_{Y\;\to \;Z}$. Similarly, there is no exact expression for the mutual information I(X;Z) in terms of I(X;Y) and I(Y;Z). However, because a DMC is a mono-channel causal channel, it follows immediately that an exact expression also exists for I(X;Z):

$$I\left(X;Z\right)=\sum_{\hat{i},k}p^{\hat{i}k}\log_{2}\left[\frac{\sum_{\hat{j}}{\bar{A}}_{\hat{i}}^{\hat{j}}B_{\hat{j}}^{k}}{p^{k}}\right].$$

Interestingly, for every *h*, Equation (11) represents a cascade of two DMCs. Therefore, an alternative structure for two causal channels in series exists, as depicted in Figure 3c. Because the data processing inequality applies to a cascade of discrete memoryless channels, the alternative structure suggests that there is a DPI for transfer entropy. In Section 3.1.4, we show that this is indeed the case.

#### 2.2.3. The Fork

A consequence of the symmetry of MI is that directed edges can be reversed, e.g., X→Y is equivalent to Y→X. For transfer entropy and causal tensors, a similar reversal is possible. Because the linear mapping of the source pmf on the destination pmf of is an expression of the Law of Total Probability, a linear transformation exists that maps the destination on the source, leading to the following definition

**Definition** **2** (Reconstruction Operator)**.** *The* ‡-*operator, or reconstruction operator, reconstructs the source distribution, conditioned of the past of the destination, from the destination distribution, conditioned of the past of the destination:*

(14) $$p_{g}^{\hat{i}}=p_{g}^{j}A_{gj}^{‡\hat{i}},$$

*with $A_{gj}^{‡\hat{i}}\;=\;p_{gj}^{\hat{i}}$. The* ‡-*operation changes the sign of the interaction delay of the original relation.*

To indicate that the destination is the input, and the source is the output, i.e., the input is reconstructed from the output, ‡ is used. The related data transmission is expressed as $X\;\leftarrow^{‡}\;Y$. Using Equation (9), it is rather straightforward to show that, from an information theory point of view, an association and its reconstructed association are equivalent, i.e., $TE_{X\to Y}=TE_{X\leftarrow^{‡}Y}$ (see Appendix A.3). This implies that a fork can be interpreted as a chain. The product rule for a chain is therefore also applicable to a fork. Assuming that the fork is the ground truth (see Figure 4d), the tensor B of the indirect association can be expressed in terms of the other causal tensors.

(15a) $$\begin{array}{cc} B_{h\hat{j}}^{k} & ={\bar{A}}_{h\hat{j}}^{‡\hat{i}}C_{h\hat{i}}^{k},\;\mathrm{with}\;{\bar{A}}_{h\hat{j}}^{‡\hat{i}}:=p_{h\hat{j}}^{g}A_{g\hat{j}}^{‡\hat{i}}, \end{array}$$

(15b) $$\begin{array}{cc} B_{g\hat{k}}^{j} & ={\bar{C}}_{g\hat{k}}^{‡\hat{i}}A_{g\hat{i}}^{j},\;\mathrm{with}\;{\bar{C}}_{g\hat{k}}^{‡\hat{i}}:=p_{g\hat{k}}^{h}C_{h\hat{k}}^{‡\hat{i}}. \end{array}$$

Equation (15a) applies in case the equivalent chain is $Y\;\to^{‡}\;X\;\to \;Z$. If the equivalent chain is $Y\;\leftarrow \;X\;\leftarrow^{‡}\;Z$, Equation (15b) is applicable. Due to the way we determine the interaction delay, the ‡-operation induces a sign change for the interaction delay. For example, if $\tau_{xy}$ represents the interaction delay for the relation X→Y, then $-\tau_{xy}$ represents the interaction delay for the relation $Y^{‡}\;\to \;X$.

#### 2.2.4. The v-Structure and the Directed Triangle

In a bivariate measurement, we will always be able to determine the ground truth correctly in the case of the v-structure depicted in Figure 4c. However, to apply the tensor formalism to structures with a collider, the v-structure and the more general directed triangle, multivariate tensors are needed. Thus, let us assume that the ground truth is the directed triangle in Figure 4d. We now have to introduce the multivariate relation D:{X,Y}→Z. This relation leads to the additional linear transformation

$$p_{h}^{k}=p_{h}^{\hat{i}\hat{j}}D_{h\hat{i}\hat{j}}^{k}.$$

We call the tensor D the *interaction tensor*. The tensors B and C can be expressed in terms of the tensor D. This follows directly from the Law of Total Probability.

(16a) $$\begin{array}{cc} B_{h\hat{j}}^{k} & ={\bar{A}}_{h\hat{j}}^{‡\hat{i}}D_{h\hat{i}\hat{j}}^{k}, \end{array}$$

(16b) $$\begin{array}{cc} C_{h\hat{i}}^{k} & ={\bar{A}}_{h\hat{i}}^{\hat{j}}D_{h\hat{i}\hat{j}}^{k}. \end{array}$$

In Appendix A.4, a proof is presented for the chain. These equations can be interpreted as representing cascades. For example, the cascade X→{X,Y}→Z consists of the inverse multiplexers represented by A and D in series, resulting in C. The tensor relations are not apparent from Figure 4c,d: these graphs do not support the calculation rules for causal tensors. In comparison, the graphs in Figure 4c,d do support the tensor relations.

**Proposition** **1.** *If a complex system contains v-structures, the causal graph must be represented by a directed hypergraph [32]. In a hypergraph, an edge connects any number of vertices. The interaction tensor corresponds to a so-called hyperedge.*

As stated in the Introduction, the approach in the article was inspired by Turing machines. The causal tensor is a realization of the transition function of a Turing machine that encodes causality in as far as the causality is encoded in the pmfs. To warrant the use of the adjective “causal”, however, it needs to be shown that, within the framework of causal tensors, we can differentiate between direct and indirect associations. That this seems possible can be intuited when considering the chain X→Y→Z. The relation X→Z is a resultant of the other relations, i.e., an indirect association. Within the framework of causal tensors, we would expect that we can express this indirect association in terms of the tensors of the other relations. In the next section, this and other consequences of the framework of causal tensors are presented.

## 3. Results

### 3.1. Differentiation Between Direct and Indirect Association using Bivariate Analysis

#### 3.1.1. A Fork Can Be Differentiated from a Chain

Let us assume that in the system comprising three variables either the chain or fork is the ground truth. In a pairwise analysis, the directed triangle in Figure 4d will be measured. In the case of a chain, Equation (11) is valid, while, in the case of a fork, Equation (15a) is valid. The question is: Can both be valid at the same time? If not, we can differentiate between a fork and a chain. Thus, let us assume that both equations are valid. Substituting the right-hand side of Equation (11) into the right-side of Equation (15a), and vice versa, leads to two new equations that must both be valid,

(17a) $$\begin{array}{cc} B_{h\hat{j}}^{k} & ={\bar{A}}_{h\hat{j}}^{‡\hat{i}}{\bar{A}}_{h\hat{i}}^{\hat{j}}B_{h\hat{j}}^{k}, \end{array}$$

(17b) $$\begin{array}{cc} C_{h\hat{i}}^{k} & ={\bar{A}}_{h\hat{i}}^{\hat{j}}{\bar{A}}_{h\hat{j}}^{‡\hat{i}}C_{h\hat{i}}^{k}. \end{array}$$

It can immediately be seen that both equations can be valid if and only if ${\bar{A}}_{h\hat{j}}^{‡\hat{i}}{\bar{A}}_{h\hat{i}}^{\hat{j}}\;=\;\delta_{\hat{j}}^{\hat{j}}$, and ${\bar{A}}_{h\hat{i}}^{\hat{j}}{\bar{A}}_{h\hat{j}}^{‡\hat{i}}\;=\;\delta_{\hat{i}}^{\hat{i}}$, with $\delta_{\hat{j}}^{\hat{j}}$ the Kronecker delta introduced above. Because the tensors are stochastic, a tensor product of two tensors can only evaluate to a Kronecker delta when each tensor has only one nonzero entry per row and column, i.e., these tensor represent a noiseless transmission. Because the tensors are themselves the product of two tensors (see Equation (A7)), these equations can only both be valid if A is a noiseless tensor, i.e., *X* and *Y* are equivalent. To differentiate between a chain and a fork, it suffices to determine if Equation (11), or Equation (15a), is valid.

#### 3.1.2. Bivariate Analysis Suffices to Infer the Structure

The tensor ${\bar{A}}_{h\hat{i}}^{\hat{j}}$ is a trivariate tensor because index *h* is related to variable *Z*, index $\hat{i}$ is related to the variable *X*, and index $\hat{j}$ is related to the variable *Y*. Thus, although the analysis is pairwise, a multivariate analysis seems to be necessary to infer the structure. However, another novel consequence of the proposed formalism is the following theorem.

**Theorem** **2** (Structure Invariance With Respect to Causal Channel Model)**.** *For a chain and a fork, the ground truth is invariant with respect to the causal channel model used, i.e., a mono-channel or multichannel causal model.*

(18) $$C_{h\hat{i}}^{k}={\bar{A}}_{h\hat{i}}^{\hat{j}}B_{h\hat{j}}^{k}\Leftrightarrow C_{\hat{i}}^{k}=A_{\hat{i}}^{\hat{j}}B_{\hat{j}}^{k}.$$

In Appendix A.5, the proof is presented. From this proof, it immediately follows that this theorem is only valid when we use the same interaction delays, and the same (applicable) embedding for both the mono-channel causal channels and the multichannel causal channels.

#### 3.1.3. A Directed Triangle Can Be Differentiated From a Chain and a Fork

In Appendix A.2, it is proven that a directed triangle can be distinguished from a chain and a fork using the causal tensors. If a structure is neither a fork nor a chain, all associations are direct. A related result is that indirect associations do not contribute to the interaction tensor. Specifically, this means that, if and only if the chain is the ground truth, $D_{h\hat{i}\hat{j}}^{k}=B_{h\hat{j}}^{k}$. This implies that, from the viewpoint of *Z*, no information should be attributed to *X*. All information that passes from *X*, via *Y*, can be considered as *shared* with *Y*.

In the case the fork is the ground truth, $D_{h\hat{i}\hat{j}}^{k}=C_{h\hat{i}}^{k}$. This implies that, again, from the viewpoint of *Z*, there is no information in *Z* that can be attributed to *Y*, but there is information in *Z* that can be attributed uniquely to *X*.

#### 3.1.4. A Data Processing Inequality Exists for TE

As shown above, there is an alternative structure for a cascade of two causal channels comprising sub-channels with cascades of DMCs (see Figure 3c). This implies that a data processing inequality exists for inverse multiplexers. In Appendix A.6, it is shown that this is indeed the case.

**Theorem** **3** (DPI for a Chain)**.** *For the chain X→Y→Z, the following inequality holds:*

(19) $$TE_{X\to Z}\;\leq \min\left[TE_{X\to Y},\;TE_{Y\to Z}\right].$$

*Because the fork has equivalent chains, the DPI also applies to a fork.*

If the proposed formalism is not used, the DPI can identify potential indirect relations. Because we do not make any assumption about the cardinality of the finite alphabet, we can, in theory, select an alphabet with any cardinality. The DPI therefore also applies to TE. The DPI for TE gives a sufficient condition to assess if a relation is a proper direct relation. It gives a necessary condition to detect potential indirect relations.

### 3.2. Examples

We finalize this article with three examples. In the first example, the structures of two distributions are determined. Apart from showing that the proposed formalism provides novel insights, it allows the readers to familiarize themselves with the concepts and application of the formalism. In the second example, the “structure” of a set of coupled differential equations is inferred. The structure reflects the temporal precedence suggested by the differential equations. In the last example, the framework is applied to a multivariate data set recorded from a patient suffering from apnea. This is the same set used in [10].

#### 3.2.1. Differentiating Between Dyadic and Triadic Distributions

The dyadic and triadic data distributions (see Table 2) have different underlying dependency structures; however, there are no conventional Shannon-like information measures that can distinguish between these two distributions [33]. We now show that modeling the data, as resulting from a communication process, results in a clear distinction between these distributions. This distinction disappears when reverting to mutual information or channel capacity. Because we use mono-channel causal channel to model the communication process, the calculations are straightforward, and can be performed manually.

First, let us determine all causal tensors for the dyadic distribution using mono-channel causal channels to model the transmission. Throughout the article, it is assumed that the pmfs are represented as row vectors, e.g., the vector (1,0,0,0) represents X=0 and (0,0,0,1) represents X=3. The rows of the transition probability matrices therefore contain the output probabilities conditioned on a specific input. For example, for the input X=0, the result of the transmission is either Y=0 or Y=2. These outcomes are equiprobable: the first row of the transition probability matrix therefore equals $\left(\frac{1}{2},0,\frac{1}{2},0\right)$. Continuing in this fashion, the reader can verify that the causal tensor for the relations X→Y, and X→Z equal

$$A=\left(\begin{array}{cccc} \frac{1}{2} & 0 & \frac{1}{2} & 0\\ \frac{1}{2} & 0 & \frac{1}{2} & 0\\ 0 & \frac{1}{2} & 0 & \frac{1}{2}\\ 0 & \frac{1}{2} & 0 & \frac{1}{2} \end{array}\right),\;C=\left(\begin{array}{cccc} \frac{1}{2} & \frac{1}{2} & 0 & 0\\ 0 & 0 & \frac{1}{2} & \frac{1}{2}\\ \frac{1}{2} & \frac{1}{2} & 0 & 0\\ 0 & 0 & \frac{1}{2} & \frac{1}{2} \end{array}\right)$$

All other tensors can be expressed in terms of these two tensors: $B\;=\;A,\;A^{‡}\;=\;C,\;B^{‡}\;=\;C$, and $C^{‡}\;=\;A$. To determine the structure of this distribution, we need to check if any of these tensors is the result of a cascade. For example, does the relation X→Z result from the cascade X→Y→Z? In total, there are six of these cascades and related tensor products to be evaluated,

(20) $$\begin{array}{cccc} \; & X\to Y\to Z: & \; & C_{i}^{k}=A_{i}^{j}B_{j}^{k}?\\ \; & Y\to Z^{‡}\to X: & \; & A_{j}^{‡i}=B_{j}^{k}C_{k}^{‡i}?\\ \; & Z^{‡}\to X\to Y: & \; & B_{k}^{‡j}=C_{k}^{‡i}A_{i}^{j}?\\ \; & Y^{‡}\to X\to Z: & \; & B_{j}^{k}=A_{j}^{‡i}C_{i}^{k}?\\ \; & X\to Z^{‡}\to Y: & \; & A_{i}^{j}=C_{i}^{k}B_{k}^{‡j}?\\ \; & Z^{‡}\to Y\to X: & \; & C_{k}^{‡i}=B_{k}^{‡j}A_{j}^{‡i}? \end{array}$$

Using the above-derived tensors, we find that none of these tensors result from a cascade. Therefore, none of the associations are indirect, and the structure is that of a (un)directed triangle.

For the triadic distribution, the causal tensors are given by:

$$A=\left(\begin{array}{cccc} \frac{1}{2} & 0 & \frac{1}{2} & 0\\ 0 & \frac{1}{2} & 0 & \frac{1}{2}\\ \frac{1}{2} & 0 & \frac{1}{2} & 0\\ 0 & \frac{1}{2} & 0 & \frac{1}{2} \end{array}\right),$$

$B\;=\;A,\;C\;=\;A,\;A^{‡}\;=\;A,\;B^{‡}\;=\;A$, and $C^{‡}\;=\;A$. All the equations on the right-hand side in Equation (20) reduce to A·A. The reader can confirm that this matrix product evaluates to A=A·A. The ground structure is that of a (very special) chain.

From this, we can conclude that the structures of the dyadic distribution and the triadic distribution are different. At the moment, we use a pointwise measure for the strength; the difference in structure is no longer apparent. For example, using the Blahut–Arimoto [34] algorithm to determine the channel capacity for these matrices, we find that the channel capacities for all relations in both distributions equal one bit.

#### 3.2.2. Coupled Ornstein–Uhlenbeck Processes

In this example, we show that the proposed formalism is capable to infer the underlying structure in a system of four coupled Ornstein–Uhlenbeck processes with independent unit variance white noise processes η [3]:

(21) $$\begin{array}{cc} \dot{x}\left(t\right) & =-0.5x\left(t\right)+0.6w\left(t-4\right)\cdot \eta_{x}\left(t\right),\\ \dot{y}\left(t\right) & =-0.9y\left(t\right)-1.0x\left(t-2\right)+0.6z\left(t-5\right)+\eta_{y}\left(t\right),\\ \dot{z}\left(t\right) & =-0.7z\left(t\right)-0.5y\left(t-6\right)+\eta_{z}\left(t\right),\\ \dot{w}\left(t\right) & =-0.8w\left(t\right)-0.4y{\left(t-3\right)}^{2}+0.05y\left(t-3\right)+\eta_{w}\left(t\right). \end{array}$$

**Figure 5:**
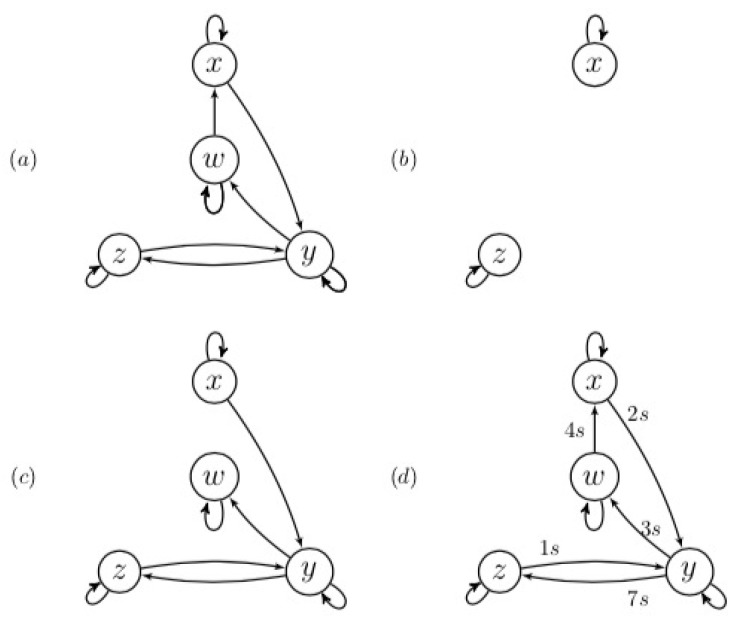
(**a**) The causal structure for the Ornstein–Uhlenbeck system of Equation (21). The other graphs show the inferred causal structures at different time series lengths. The confidence interval was 90% and the maximum delay was set to 20s: (**b**) *T* = 10k s and (**c**) *T* = 100k s. In (**d**), *T* = 500k s, the interaction delays that maximized the channel capacity are also shown.

The directionality between variables follows from the time delays in the differential equations, i.e., *temporal precedence*. The data were generated using an integration time step of dt = 0.01 s, and a sampling interval of Δs = 100 s. For demonstration purposes, we used a binary encoding scheme. First, the data were normalized, after which they were partitioned at 0.5. The *y* embedding was set to one, ℓ=1, and the *x* embedding was set to zero, m=0; therefore, each sub-channel consists of a special DMC, the binary asymmetric channel, for which a closed expression for the channel capacity exists [35]. Binary encoding reduces the information content between time series, resulting in weak causal channels, reducing the probability of detecting an indirect relation, as illustrated by Figure 5d, where no pruning was required. Because of the reduced information content, three datasets were used for this experiment: a dataset comprising 10k samples, a dataset comprising 100k samples, and a dataset comprising 500k samples. Per data pair, the optimal interaction delay was determined by changing the assumed interaction delay for 0–20 s in steps of 1 s. Per assumed interaction delay the existence of an edge is determined: if the difference between the two channel capacities differs significantly from zero, an edge exists for the pair with the largest channel capacity. Next, per cause/effect pair, the interaction delay is chosen that maximizes the channel capacity.

Because the data are binary, Jeffrey’s interval estimation for a binomial proportion was used to determine the confidence intervals [36]; in this example, a confidence interval of 90% was used. The calculations were performed on a standard-issue laptop comprising an Intel i5-6300U CPU @2.40GHz, and 8Gb RAM. The proposed framework was implemented in MATLAB R2018b. The processing times were, respectively, 1.16 s for the 10k dataset, 3.30 s for the 100k dataset, and 16.18 s for the 500k dataset.

As can be seen in Figure 5, the interaction delays are close, or equal to the interaction delays in the differential equations. Only the interaction delay for the relation z→y shows a large difference, i.e., $\tau_{zy}=1$ s instead of 5 s. Furthermore, the framework can correctly identify cycles between a pair of variables.

#### 3.2.3. 1991 Santa Fe Time Series Competition Data Set B

In the last example, the framework is applied to a multivariate data set recorded from a patient suffering from sleep apnea. The dataset consists of 34k samples of three variables, heart rate, respiration force, and the blood oxygen concentration, measured while the patient was (intermittent) sleeping. The sampling frequency was 2 Hz [37]. Apart from these variables, the times and stages of sleep were recorded [38]. The goal of this example is twofold: (1) compare with the method in [10], in which it was found that the transfer entropy from heart rate towards respiration force is larger than the transfer entropy from respiration force towards heart rate; and (2) determine if the underlying structure while the patient was sleeping (the wake/sleep stages) differs from the underlying structure while the patient was awake. This resulted in two datasets, one comprising the 19.7k samples during the awake stages, and the other comprising the 14.8k samples taken during the wake/sleep stages.

Again, a binary encoding scheme was used, $\tilde{x}=0$ unless x(t+1)≥x(t), in which case $\tilde{x}=1$. The *y* embedding was set to one (ℓ=1) for all variables and the *x* embedding was set to zero (m=0). The heart rate and respiration force can be modeled as a first order Markov chain. The order of the Markov chain for the blood oxygen concentration is higher, but as we are interested in differences, we still chose ℓ=1. Per data pair, the optimal interaction delay was determined over a range of 100 samples (50 s) using the average over the channel capacities per sub-channel instead of transfer entropy. Typical processing times per dataset was 1.6 s.

**Figure 6:**
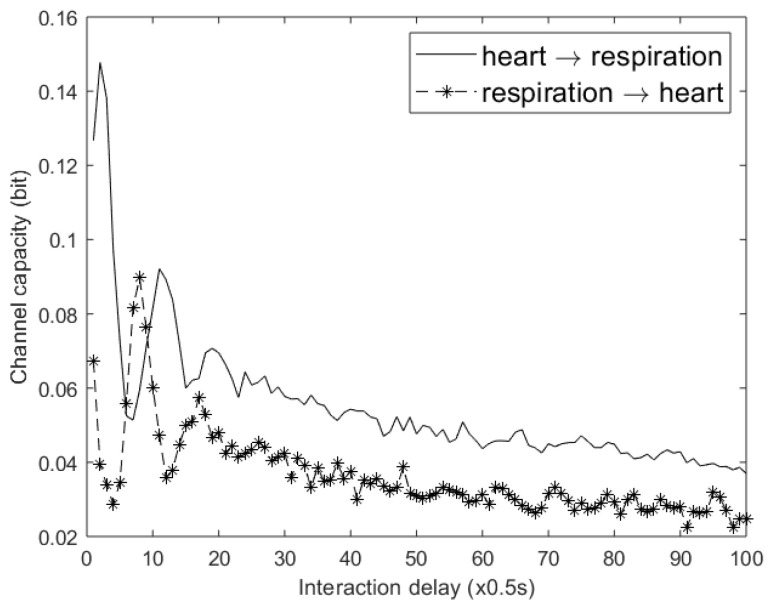
The channel capacity as a function of the assumed interaction delay for the pairs heart→respiration and respiration→heart during the wake/sleep stages of the patient.

**Figure 7:**
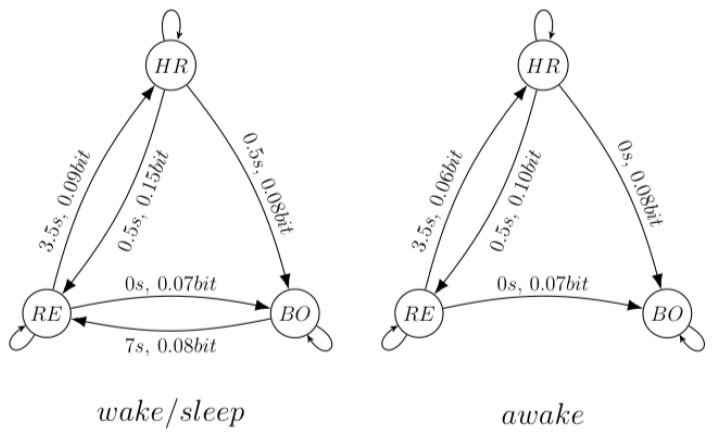
The underlying structures at a confidence interval of 99% for the data sets representing the wake/sleep stages and the awake stage. HR is the heart rate, RE the respiration force and BO the blood oxygen concentration. The channel capacities (bit) and interaction delays (seconds) are also indicated. The interaction delays of the “self-influences” were 4.5 s for the heart rate, 3.5 s for the respiration force, and 1.5 s for the blood oxygen concentration.

Using the procedure as described in Figure 6, a significant difference was found in the underlying structures between the patients awake stage and the patients wake/sleep stage (see Figure 7).

This example shows that, given the naïve selection of the embedding, in accordance with Schreiber [10], the relation heart→respiration is stronger than the relation respiration→heart. Additionally, a difference in the underlying structure between the awake and the wake/sleep stages was found: during the latter stages, the blood oxygen concentration seems to influence the respiration force, with a typical interaction delay of 7 s. This example should be interpreted as a demonstration of the framework on real data, not as a full blown analysis of this specific dataset.

## 4. Discussion

In this article, we focus on establishing the basic foundations, not on providing a complete framework of a tensor based approach to infer causal structures from time series. Although it uses TE as a starting point, it leads to new insights that cannot be reached with TE. For example, the formalism allows us to derive previously non-existent expressions for cascades, and it is able to detect differences in underlying structure that cannot be detected by TE or MI. To turn this formalism into a useable addition to the causal inference toolset, some fundamental questions need to be addressed in future work. How can temporal information be incorporated in the formalism? Can this formalism be applied to systems comprising more than three variables? How can it be made applicable to non-stationary data? Furthermore, related to the last question and computational issues, what is the impact of different data encoding strategies on computational costs and accuracy of the inferred structures?

Although there is still a lot of work to be done, and apart from the novel insights thus far, the formalism might open two new avenues of research: (1) Once the structure has been determined, the behavior of the system can be simulated because the transformation rules are encoded in the causal tensors. (2) Because of the novel insights that were achieved using the proposed formalism thus far, it might contribute to the “Partial Information Decomposition’’ discussion [39]. The way our formalism might contribute is by using its capability of determining exact expressions for indirect paths (paths comprising two or more edges). Combined with the observations resulting from investigating the interaction tensor in the case of the chain and the fork, redundancy and uniqueness of information is related to indirect paths and direct paths, respectively, our approach will be focused on definitions of redundancy and uniqueness in terms of direct and indirect paths.

## Figures and Tables

**Table 1:** Overview of indices used.

Process	Variable	Alphabet Element	Index (Input)	Index (Past)	Index (Output)
*X*	*x*	χ	$\hat{i}$	*f*	*i*
*Y*	*y*	ψ	$\hat{j}$	*g*	*j*
*Z*	*z*	ζ	$\hat{k}$	*h*	*k*

**Table 2:** Two systems, both comprising three random variables with identical joint probabilities per combination of the random variables. The underlying structures are very different, which can be seen when the variables are represented in two bits, e.g., the binary expansion for X=3 equals $X_{0}X_{1}\;=\;11$. (**a**) For the dyadic (pairwise) set, $X_{0}\;=\;Y_{1}$,$Y_{0}\;=\;Z_{1}$, and $Z_{0}\;=\;X_{1}$. (**b**) For the triadic (three-way) set, $X_{0}+Y_{0}+Z_{0}$ mod2, and $X_{1}+Y_{1}+Z_{1}$.

(a) Dyadic				(b) Triadic			
*X*	*Y*	*Z*	*p*	*X*	*Y*	*Z*	*p*
0	0	0	$\frac{1}{8}$	0	0	0	$\frac{1}{8}$
0	2	1	$\frac{1}{8}$	1	1	1	$\frac{1}{8}$
1	0	2	$\frac{1}{8}$	0	2	2	$\frac{1}{8}$
1	2	3	$\frac{1}{8}$	1	3	3	$\frac{1}{8}$
2	1	0	$\frac{1}{8}$	2	0	2	$\frac{1}{8}$
2	3	1	$\frac{1}{8}$	3	1	3	$\frac{1}{8}$
3	1	2	$\frac{1}{8}$	2	2	0	$\frac{1}{8}$
3	3	3	$\frac{1}{8}$	3	3	1	$\frac{1}{8}$

**Figure A1:**
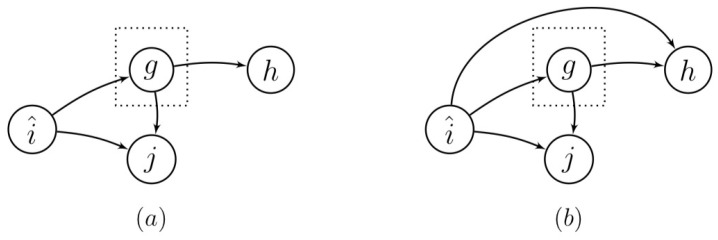
Illustration of the Causal Markov Condition. (**a**) The vertex in the dotted box blocks all paths between $\hat{i}$ and *h*, and between *j* and *h*. According to the Causal Markov Condition, $\left\{\hat{i},j\right\}$ and *h* are independent given *g*: $p\left(\hat{i},j,h|g\right)\;=\;p\left(\hat{i},j|g\right)p\left(h|g\right)$. (**b**) The vertex in the dotted box does not block all path between $\hat{i}$ and *h*, and between *j* and *h*. According to the Causal Markov Condition, $\left\{\hat{i},j\right\}$ and *h* are *not* independent given *g*, i.e., $p\left(\hat{i},j,h|g\right)\;\neq \;p\left(\hat{i},j|g\right)p\left(h|g\right)$.

**Figure A2:**
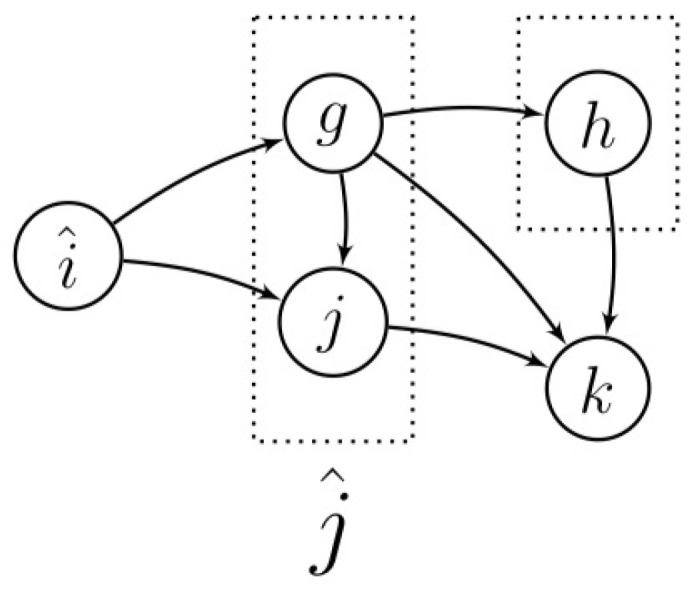
The graph related a chain of two causal channels. Both dotted boxes block the paths between $\hat{i}$ and *k*. The dotted box comprising the variables {g,j} also blocks all paths between $\hat{i}$ and *k*. This box represents the variable $\hat{j}$.

## Abbreviations

The following abbreviations are used in this manuscript:

DMC	discrete memoryless communication channel
DPI	data processing inequality
MI	mutual information
pmf	probability mass function
TE	transfer entropy

## Appendix A. Overview and Proofs

In this appendix, an overview is given of the used definitions, theorems and proofs relevant to the formalism.

**Proposition** **A1.** *In the case MI or MI related measures are used to infer the* ***structure*** *for a system, we should use the probability transition tensors or measures based on elements of probability transition tensor.*

### Appendix A.1. Causal Channel

**Definition** **A1** (Causal Channel)**.** *A causal channel is an inverse multiplexer in which the demultiplexer selects the sub-channel over which the data are sent based on the past output data. Each sub-channel comprises a DMC. The input symbol is fed to a specific input vertex of the chosen discrete memoryless channel. The DMC transforms the input in a probabilistic fashion into an output symbol. The multiplexer combines the outputted symbols into the output message.*

**Theorem** **A1** (TE results from data transmission over an inverse multiplexer)**.** *Transfer entropy is the average conditional mutual information of transmission over a causal channel.*

**Proof.** The relative frequency with which the *g*th sub-channel is chosen equals $p(\psi_{g}^{-})$. Each sub-channel is a DMC, thus the mutual information of the *g*th sub-channel equals $I(X;Y|\psi_{g}^{-})$. The weighted average of the mutual information over all the sub-channels is equal to $\sum_{\psi_{g}^{-}\in Y^{\ell }}p\left(\psi_{g}^{-}\right)I\left(X;Y|\psi_{g}^{-}\right)$, which is the definition of TE in Equation (7). □

### Appendix A.2. The Chain

**Theorem** **A2** (Product Rule for a Chain)**.** *Let A and B be the causal tensors of two causal channels in series. Let the tensor C represent the resulting indirect causal channel that must be measured in a bivariate approach. The tensor elements of C are given by*

(A1) $$C_{h\hat{i}}^{k}=p_{h\hat{i}}^{g}A_{g\hat{i}}^{\hat{j}}B_{h\hat{j}}^{k}.$$

*If the ground truth is a directed triangle, the product rule for a chain is invalid, i.e., $C_{h\hat{i}}^{k}\;\neq \;p_{h\hat{i}}^{g}A_{g\hat{i}}^{\hat{j}}B_{h\hat{j}}^{k}$.*

For the proof of Theorem A2, we need to introduce two lemmas.

**Lemma** **A1.**
*$\forall \;g:\;B_{gh\hat{j}}^{k}=B_{h\hat{j}}^{k}$.*

**Lemma** **A2.**
*For the chain X→Y→Z, $A_{\hat{i}gh}^{\hat{j}}=A_{\hat{i}g}^{\hat{j}}$.*

**Proof** **for** **Lemma** **A1.** A direct consequence of the Markov property is related to indices associated with the same random variable. As long as the index related to the past of the output (*g*) and the index related to the output (*j*) appear in the same tensor, we are allowed to replace the output index by the input index. In our example, this means that we may replace *j* by $\hat{j}$ as long as we ensure that $\psi_{\hat{j}}^{-}=\left\{\psi_{j},\psi_{g}^{-}\right\}$. This is always possible because of the Markov property: we enlarge the cardinality of either $\psi_{\hat{j}}^{-}$ or $\psi_{g}^{-}$. □

**Proof** **for** **Lemma** **A2.** Figure A1a depicts the situation of the chain X→Y→Z. According to the Causal Markov Condition, $\left\{\hat{i},j\right\}$ and *h* are independent given *g*, i.e.,

(A2) $$p\left(\hat{i},j,h|g\right)\;=\;p\left(\hat{i},j|g\right)p\left(h|g\right).$$

The left-hand side can be rewritten as $p\left(\hat{i},j,h|g\right)\;=\;p\left(j,h|\hat{i},g\right)p\left(\hat{i}|g\right)$. The right-hand side is rewritten using Figure A1a again. According to the Causal Markov Condition, $\hat{i}$ and *j* are independent given *g*: $p\left(\hat{i},j|g\right)\;=\;p\left(j|\hat{i},g\right)p\left(\hat{i}|g\right)$. This finally leads the conclusion that Equation (A2) equals

$$p\left(j|\hat{i},g,h\right)=p\left(j|\hat{i},g\right),$$

i.e., $A_{\hat{i}gh}^{\hat{j}}=A_{\hat{i}g}^{\hat{j}}$. □

**Proof** **for** **Theorem** **A2.** Because it is a straightforward exercise in which we again make use of the Law of Total Probability, we leave it to the reader to confirm that

(A3a) $$\begin{array}{cc} p_{g}^{j} & =p_{g}^{\hat{i}}A_{g\hat{i}}^{j}\;, \end{array}$$

(A3b) $$\begin{array}{cc} p_{h}^{k} & =p_{h}^{\hat{j}}B_{h\hat{j}}^{k}\;, \end{array}$$

(A3c) $$\begin{array}{cc} p_{h}^{k} & =p_{h}^{\hat{i\prime }}C_{h\hat{i\prime }}^{k}\;. \end{array}$$

The index $\hat{i\prime }$ in Equation (A3c) represents a different input vector than the index $\hat{i}$ in Equation (A3a), although they both refer to the random variable *X*. This is because $\hat{i\prime }$ is related to the source $x\prime^{-}\in X^{m\prime }$ of *Z* and $\hat{i}$ is the index related to the source $x^{-}\in X^{m}$ of *Y*. The Markov property however immediately implies that we can use one and the same index in both cases if we select the source vector with the largest cardinality. Without loss of generality, we use $\hat{i}$. Because of the Law of Total Probability, we are allowed to condition Equation (A3a) on *h* and Equations (A3b) and (A3c) on *g*. This leads to

(A4a) $$\begin{array}{cc} p_{gh}^{\hat{j}} & =p_{gh}^{\hat{i}}A_{gh\hat{i}}^{\hat{j}}, \end{array}$$

(A4b) $$\begin{array}{cc} p_{gh}^{k} & =p_{gh}^{\hat{j}}B_{gh\hat{j}}^{k}, \end{array}$$

(A4c) $$\begin{array}{cc} p_{gh}^{k} & =p_{gh}^{\hat{i}}C_{gh\hat{i}}^{k}. \end{array}$$

Substituting the expression for $p_{gh}^{\hat{j}}$ of Equation (A4a) into Equation (A4b) and combining the result with Equation (A4c) gives us $C_{gh\hat{i}}^{k}=A_{gh\hat{i}}^{\hat{j}}B_{gh\hat{j}}^{k}$. Using Lemmas A1 and A2, this can be rewritten as

(A5) $$C_{gh\hat{i}}^{k}=A_{g\hat{i}}^{\hat{j}}B_{h\hat{j}}^{k}.$$

Finally, we multiply both sides with $p_{h\hat{i}}^{g}$. As the reader can confirm, the term $p_{h\hat{i}}^{g}C_{gh\hat{i}}^{k}$ equals $C_{h\hat{i}}^{k}$. This finally leads to Equation (A1). For the second part of the theorem, we refer to Figure 2b. It depicts the situation of the directed triangle. Apart from the chain X→Y→Z, the relation X→Z exists. According to the Causal Markov Condition, $\left\{\hat{i},j\right\}$ and *h* are *not* independent given *g*: $p\left(\hat{i},j,h|g\right)\;\neq \;p\left(\hat{i},j|g\right)p\left(h|g\right)$. □

**Lemma** **A3.**
*For a chain, the product $p_{h\hat{i}}^{g}A_{g\hat{i}}^{\hat{j}}$ is a stochastic tensor ${\bar{A}}_{h\hat{i}}^{\hat{j}}$.*

**Proof** **for** **Lemma** **A3.** By definition

(A6) $$p_{h\hat{i}}^{g}A_{g\hat{i}}^{\hat{j}}=\sum_{g}p\left(g|h,\hat{i}\right)p\left(\hat{j}|g,\hat{i}\right).$$

Because $\hat{j}=\left\{j,g\right\}$, $p\left(\hat{j}|g,\hat{i}\right)=p\left(j|g,\hat{i}\right)$. We can now use Lemma A2. The expression $p\left(j|\hat{i},g,h\right)=p\left(j|\hat{i},g\right)$ implies that $p\left(g|h,\hat{i}\right)p\left(j|g,\hat{i}\right)=p\left(j,g|h,\hat{i}\right)$. With this, Equation (A7) is rewritten as

(A7) $$p_{h\hat{i}}^{g}A_{g\hat{i}}^{\hat{j}}=\sum_{g}p\left(j,g|h,\hat{i}\right).$$

Applying the Law of Total Probability to the right-hand side gives us $\sum_{g}p\left(j,g|h,\hat{i}\right)=p\left(j|h,\hat{i}\right)$. In other words, $p_{h\hat{i}}^{g}A_{g\hat{i}}^{\hat{j}}$ is a stochastic tensor. □

### Appendix A.3. Equivalence of a Fork and a Chain

**Definition** **A2** (Reconstruction Operator)**.** *The* ‡-*operator, or reconstruction operator, reconstructs the source distribution, conditioned of the past of the destination, from the destination distribution, conditioned of the past of the destination:*

(A8) $$p_{g}^{\hat{i}}=p_{g}^{j}A_{gj}^{‡\hat{i}},$$

*with $A_{gj}^{‡\hat{i}}\;=\;p_{gj}^{\hat{i}}$. The* ‡-*operation changes the sign of the interaction delay of the original relation.*

**Corollary** **A1** (Equivalence of an association and its reconstructed association)**.** *From an information theory point of view, an association and its reconstructed association are equivalent, i.e., $TE_{X\to Y}=TE_{X\leftarrow^{‡}Y}$.*

**Proof.** We first notice that $p_{g\hat{i}}^{j}\;=\;A_{g\hat{i}}^{j}$, which implies that, using the normal notation instead of the Einstein summation convention, $p_{g}^{\hat{i}j}\;=\;p_{g}^{\hat{i}}A_{g\hat{i}}^{j}$. Equivalently, $p_{gj}^{\hat{i}}\;=\;A_{gj}^{‡\hat{i}}$ implies that $p_{g}^{\hat{i}j}\;=\;p_{g}^{j}A_{gj}^{‡\hat{i}}$. Combining both equations lead to $A_{g\hat{i}}^{j}/p_{g}^{j}\;=\;A_{gj}^{‡\hat{i}}/p_{g}^{\hat{i}}$. Combining this with Equation (9) results in $TE_{X\to Y}=TE_{X\leftarrow^{‡}Y}$. □

### Appendix A.4. The Interaction Tensor

**Lemma** **A4** (Causal Tensor Contraction)**.** *In the case of a directed triangle, we can express the causal tensors in terms of the interaction tensor:*

(A9a) $$\begin{array}{cc} B_{h\hat{j}}^{k} & ={\bar{A}}_{h\hat{j}}^{‡\hat{i}}D_{h\hat{i}\hat{j}}^{k}, \end{array}$$

(A9b) $$\begin{array}{cc} C_{h\hat{i}}^{k} & ={\bar{A}}_{h\hat{i}}^{\hat{j}}D_{h\hat{i}\hat{j}}^{k}. \end{array}$$

Because of the equivalence of a chain and a fork, we only prove the lemma for a chain.

**Proof** **for** **Lemma** **A4.** First, we note that $p_{h}^{\hat{i}\hat{j}}\;=\;\delta_{\hat{j}}^{\hat{j\prime }}p_{h}^{\hat{j}}p_{h\hat{j}\prime }^{\hat{i}}$. Equation (2.2.4) can therefore be rewritten as $p_{h}^{k}=\delta_{\hat{j}}^{\hat{j\prime }}p_{h}^{\hat{j}}p_{h\hat{j}\prime }^{\hat{i}}D_{h\hat{i}\hat{j}}^{k}$. Because we are allowed to change the order of $\delta_{\hat{j}}^{\hat{j\prime }}$ and $p_{h}^{\hat{j}}$, we get $p_{h}^{k}\;=\;p_{h}^{\hat{j}}\left(\delta_{\hat{j}}^{\hat{j\prime }}p_{h\hat{j}\prime }^{\hat{i}}D_{h\hat{i}\hat{j}}^{k}\right)$. Combining this with Equation (A3b) results in an expression for $B_{h\hat{j}}^{k}$: $B_{h\hat{j}}^{k}=\delta_{\hat{j}}^{\hat{j\prime }}p_{h\hat{j}\prime }^{\hat{i}}D_{h\hat{i}\hat{j}}^{k}$. Because $\delta_{\hat{j}}^{\hat{j\prime }}p_{h\hat{j}\prime }^{\hat{i}}\;=\;p_{h\hat{j}}^{\hat{i}}$, we get Equation (A9a). □

### Appendix A.5. Bivariate Analysis Suffices

**Theorem** **A3** (Structure Invariance With Respect to Causal Channel Model)**.** *For a chain and a fork, the ground truth is invariant with respect to the causal channel model used, i.e., a mono-channel or multichannel causal model.*

(A10) $$C_{h\hat{i}}^{k}={\bar{A}}_{h\hat{i}}^{\hat{j}}B_{h\hat{j}}^{k}\Leftrightarrow C_{\hat{i}}^{k}=A_{\hat{i}}^{\hat{j}}B_{\hat{j}}^{k}.$$

The proof for Theorem 2 uses Figure A2. We furthermore do not use the Einstein summation convention.

**Proof** **of** **Theorem** **2.** First, we prove that $C_{h\hat{i}}^{k}=\sum_{\hat{j}}{\bar{A}}_{h\hat{i}}^{\hat{j}}B_{h\hat{j}}^{k}\Rightarrow C_{\hat{i}}^{k}=\sum_{\hat{j}}A_{\hat{i}}^{\hat{j}}B_{\hat{j}}^{k}$. According to the Law of Total Probability, $C_{\hat{i}}^{k}\;=\;\sum_{h}p_{\hat{i}}^{h}C_{h\hat{i}}^{k}$. Multiplying both sides of of the left-hand side of the implication by $p_{\hat{i}}^{h}$ results in

(A11) $$C_{\hat{i}}^{k}=\sum_{\hat{j}}\sum_{h}p_{\hat{i}}^{h}{\bar{A}}_{h\hat{i}}^{\hat{j}}B_{h\hat{j}}^{k}.$$

We now express all stochastic tensors with the letter *p* instead of an *A* or a *B*. Equation (A11) is equivalent to

(A12) $$C_{\hat{i}}^{k}=\sum_{\hat{j}}p_{\hat{i}}^{\hat{j}}p_{\hat{j}}^{k}\sum_{h}\frac{p_{\hat{i}}^{h}p_{h\hat{i}}^{\hat{j}}p_{h\hat{j}}^{k}}{p_{\hat{i}}^{\hat{j}}p_{\hat{j}}^{k}}.$$

Using Bayes theorem [22]: $p_{\hat{i}}^{h}p_{h\hat{i}}^{\hat{j}}\;=\;p_{\hat{i}}^{h\hat{j}}$, $p_{\hat{i}}^{h\hat{j}}/p_{\hat{i}}^{\hat{j}}\;=\;p_{\hat{j}}^{h\hat{i}}/p_{\hat{j}}^{\hat{i}}$, and $p_{\hat{j}}^{h\hat{i}}\;=\;p_{h\hat{j}}^{\hat{i}}p_{\hat{j}}^{h}$, it follows that Equation (A12) is equivalent to

(A13) $$C_{\hat{i}}^{k}=\sum_{\hat{j}}p_{\hat{i}}^{\hat{j}}p_{\hat{j}}^{k}\sum_{h}\frac{p_{h\hat{j}}^{\hat{i}}}{p_{\hat{j}}^{\hat{i}}}\frac{p_{h\hat{j}}^{k}}{p_{\hat{j}}^{k}}p_{\hat{j}}^{h}.$$

Next, the Causal Markov Condition is applied to the graph in Figure A2, giving us $\hat{i}⊥\;⊥k|\left\{h,\hat{j}\right\}$, i.e., $p_{h\hat{j}}^{\hat{i}}p_{h\hat{j}}^{k}p_{\hat{j}}^{h}\;=\;p_{\hat{j}}^{h\hat{i}k}$. Because we sum over *h*, we sum out this variable as per Law of Total Probability. Using $A_{\hat{i}}^{\hat{j}}=p_{\hat{i}}^{\hat{j}}$ and $B_{\hat{j}}^{k}=p_{\hat{j}}^{k}$, we finally get the following expression

(A14) $$C_{\hat{i}}^{k}=\sum_{\hat{j}}A_{\hat{i}}^{\hat{j}}B_{\hat{j}}^{k}\frac{p_{\hat{j}}^{\hat{i}k}}{p_{\hat{j}}^{\hat{i}}p_{\hat{j}}^{k}}.$$

Thus, if $\hat{i}$ and *k* are independent given $\hat{j}$, the theorem has been proven. When we apply the Causal Markov Condition to the graph in Figure A2, we see that $\hat{i}⊥\;⊥k|\hat{j}$. The proof fully hinges on the conditional independencies resulting from the graph in Figure A2; all other steps result in equivalent expressions due to the application of Bayes theorem and the Law of Total Probability. To prove that $C_{h\hat{i}}^{k}={\bar{A}}_{h\hat{i}}^{\hat{j}}B_{h\hat{j}}^{k}⇐C_{\hat{i}}^{k}=A_{\hat{i}}^{\hat{j}}B_{\hat{j}}^{k}$, we have take the reverse steps and start with $C_{\hat{i}}^{k}=\sum_{\hat{j}}A_{\hat{i}}^{\hat{j}}B_{\hat{j}}^{k}$. Because the structure is a chain, Equation (A14) is valid again. Now, if we ensure that $\hat{j}$ is chosen large enough, so that it blocks all paths from $\hat{i}$ to *h*, Equation (A13) is valid. Due the aforementioned equivalences, this suffices to prove that a chain in a mono-channel causal model is also a chain in a multichannel causal model. □

### Appendix A.6. The DPI

For the proof of the data processing inequality, Theorem 3, a simplified notation for transfer entropy and mutual information is used: $TE_{X\to Y}\;:=\;TE\left(A,\cdot \right),\;TE_{Y\to Z}\;:=\;TE\left(B,\cdot \right),\;TE_{X\to Z}\;:=\;TE\left(C,\cdot \right)$ and $I\left(X;Y\right)\;:=\;I\left(A_{h},\cdot \right),\;I\left(Y;Z\right)\;:=\;I\left(B_{h},\cdot \right),\;I\left(X;Z\right)\;:=\;I\left(C_{h},\cdot \right)$. The subscript *h* indicates the *h*th sub-channel representing a DMC.

**Proof** **for** **Theorem** **3.** We start with Equation (11). The DPI is valid per sub-channel. Thus, for all *h*: $I\left(C_{h},\cdot \right)\leq \min\left[I\left(A_{h},\cdot \right),I\left(B_{h},\cdot \right)\right]$. As per Equation (7), we multiply both sides by $p(\zeta_{h}^{-})$—the probability that the *h*th channel is selected—and sum over *h*. This results in a DPI for transfer entropy,

(A15) $$TE\left(C,\cdot \right)\;\leq \;\min[TE\left(\bar{A},\cdot \right),TE\left(B,\cdot \right)].$$

The tensor ${\bar{A}}_{h}$ is itself the result of two cascaded channels represented by $A_{g}$ and a tensor with elements $p_{\hat{i}h}^{g}$. For these two DMC’s the DPI is also valid, leading to:

$$\forall_{g,h}:\;I\left({\bar{A}}_{h},\cdot \right)\leq I\left(A_{g},\cdot \right).$$

We now multiply both sides of this equation by $p\left(\zeta_{h}^{-}\right)p\left(\psi_{g}^{-}\right)$, and sum over *h* and *g*, resulting in $TE\left(\bar{A},\cdot \right)\leq TE\left(A,\cdot \right)$. We can now rewrite Equation (A15) as

(A16) TE(C,·)≤min[TE(A,·),TE(B,·)].

□
